# Hydrogen sulfide modulates cadmium-induced physiological and biochemical responses to alleviate cadmium toxicity in rice

**DOI:** 10.1038/srep14078

**Published:** 2015-09-11

**Authors:** Mohammad Golam Mostofa, Anisur Rahman, Md. Mesbah Uddin Ansary, Ayaka Watanabe, Masayuki Fujita, Lam-Son Phan Tran

**Affiliations:** 1 Laboratory of Plant Stress Responses, Department of Applied Biological Science, Faculty of Agriculture, Kagawa University, Miki, Kagawa 761-0795, Japan; 2 Department of Biochemistry and Molecular Biology, Bangabandhu Sheikh Mujibur Rahman Agricultural University, Gazipur 1706, Bangladesh; 3 Department of Biochemistry and Molecular Biology, Jahangirnagar University, Savar, Dhaka 1342, Bangladesh; 4 Signaling Pathway Research Unit, RIKEN Center for Sustainable Resource Science, 1-7-22, Suehiro-cho, Tsurumi, Yokohama 230-0045, Japan

## Abstract

We investigated the physiological and biochemical mechanisms by which H_2_S mitigates the cadmium stress in rice. Results revealed that cadmium exposure resulted in growth inhibition and biomass reduction, which is correlated with the increased uptake of cadmium and depletion of the photosynthetic pigments, leaf water contents, essential minerals, water-soluble proteins, and enzymatic and non-enzymatic antioxidants. Excessive cadmium also potentiated its toxicity by inducing oxidative stress, as evidenced by increased levels of superoxide, hydrogen peroxide, methylglyoxal and malondialdehyde. However, elevating endogenous H_2_S level improved physiological and biochemical attributes, which was clearly observed in the growth and phenotypes of H_2_S-treated rice plants under cadmium stress. H_2_S reduced cadmium-induced oxidative stress, particularly by enhancing redox status and the activities of reactive oxygen species and methylglyoxal detoxifying enzymes. Notably, H_2_S maintained cadmium and mineral homeostases in roots and leaves of cadmium-stressed plants. By contrast, adding H_2_S-scavenger hypotaurine abolished the beneficial effect of H_2_S, further strengthening the clear role of H_2_S in alleviating cadmium toxicity in rice. Collectively, our findings provide an insight into H_2_S-induced protective mechanisms of rice exposed to cadmium stress, thus proposing H_2_S as a potential candidate for managing toxicity of cadmium, and perhaps other heavy metals, in rice and other crops.

Cadmium (Cd) is a potential and persistent environmental contaminant, causing serious toxicity to all living organisms, including humans and plants^1^,^2^,^3^. Cd has been ranked 7^th^ among the top 20 toxins and considered human carcinogen^1^. In many South and East Asian countries, including Bangladesh, India, Japan, Indonesia and Thailand, Cd accumulation in rice and its subsequent transfer to the human food chain is a major environmental issue^2^. Rice contributed to the 36–50% of the total oral intake of Cd for Japanese population during 1998–2001^3^. In Bangladesh, rice cultivating lands adjacent to the industrial establishments are highly contaminated with Cd that was found to be between 134 and 156 mg Cd Kg^−1^ of soil^4^. Thus, preventing Cd uptake in rice plants grown in Cd-contaminated soils has become an urgent task to ensure food safety.

Cd, a non-redox water soluble heavy metal, can be quickly taken up by plant roots, and transported to the aerial parts where it significantly impedes vital cellular processes, including respiration and CO_2_ fixation^1^,^5^. Chlorosis, necrosis, epinasty, stunted growth, cell death and disturbance in mineral homeostasis are the common consequences of Cd toxicity in plants^5^,^6^,^7^. At the cellular level, Cd can bind to sulfhydryl and carbonyl groups of proteins and can replace essential cofactors, resulting in enzyme inactivation and production of reactive oxygen species (ROS), leading to oxidative stress induced damage^1^,^6^. Additionally, Cd is known to induce the production of methylglyoxal (MG), an extremely reactive aldehyde that can modify proteins, nucleic acids and carbohydrates by forming cross linkages^8^,^9^. Moreover, MG in excess can further intensify ROS production by inactivating antioxidant enzymes or by interfering with photosynthesis^8^,^10^,^11^.

Plants have developed several strategies to counteract Cd toxicity, among which sulfur-induced defense is of paramount importance^1^,^12^. Sulfur-rich small ligands, such as cysteine, glutathione (GSH) and phytochelatins, directly bind with Cd and sequestrate it into vacuoles^13^,^14^. In addition, plant cells employ a number of GSH-based antioxidative reactions to scavenge Cd-induced superoxide (O_2_^•^^−^), hydrogen peroxide (H_2_O_2_) and hydroxyl radical (OH^•^)^8^,^15^. GSH, with the help of ascorbate (AsA), plays central role in operating the AsA-GSH cycle in which ascorbate peroxidase (APX) detoxifies H_2_O_2_ in coordination to monodehydroascorbate reductase (MDHAR), dehydroascorbate reductase (DHAR) and glutathione reductase (GR)^16^,^17^. Glutathione peroxidase (GPX) and glutathione *S*-transferase (GST) also detoxify H_2_O_2_, lipid hydoperoxides and reactive aldehydes by conjugating them with GSH^18^. Likewise, plant cells eliminate MG via glyoxalase (Gly) pathway by which Gly I and Gly II enzymes act coordinately with GSH^8^,^9^. However, it has been reported that Cd-overloads deplete cytosolic GSH which impinges GSH/GSSG redox potential, causing enhanced Cd sensitivity in several plant species^19^,^20^,^21^.

Hydrogen sulfide (H_2_S) has traditionally been thought of a phytotoxin but recently, it has emerged as an important gasotransmitter alongside with nitric oxide and carbon dioxide with multiple functions in animals and plants^22^. H_2_S, at tiny doses, can enhance plant growth and development, leading to a sharp increase in global food supplies and plentiful stock for biofuel production^23^. Despite enormous prospects of H_2_S in sustainable plant agriculture, few studies have focused on H_2_S biology in plant systems compared with those in animals.

H_2_S plays multiple roles in regulating key physiological processes, such as root organogenesis^24^, photosynthesis^25^, seed germination^23^ and stomatal apertures^26^. In fact, H_2_S has received increasing attentions due to its role in orchestrating plant responses to various environmental cues^22^,^27^,^28^. Treatment with H_2_S donor sodium hydrosulfide (NaHS) improved plant tolerance to various abiotic stresses including heavy metals in wheat^29^, cucumber^30^, barley^31^ and brassica^32^. However, mechanisms regulating H_2_S functions in plant responses to heavy metal stress, especially in a major crop like rice, are still elusive.

Thus, in the current study, we have examined the regulatory role of H_2_S in rice tolerance to Cd stress. Towards this objective, a combined pharmacological, physiological, histochemical and biochemical approach was employed to identify H_2_S-controlled Cd stress tolerance mechanisms by assessing the following key components, including (i) plant growth and biomass, (ii) Cd uptake and accumulation, (iii) mineral homeostases, (iv) excessive Cd-induced oxidative stress in terms of ROS and MG levels, and lipid peroxidation, (v) photosynthesis, (vi) ascorbate, glutathione contents, and redox homeostases and (vii) the activities of the enzymes of the antioxidant defense and Gly systems in economically important rice plants under Cd stress. Additionally, this study focused on the relationship between H_2_S-induced GSH homeostases and the activities of GSH-metabolizing enzymes to evaluate their roles in alleviating Cd stress-triggered adverse effects in rice plants.

## Results

### H_2_S suppresses Cd uptake and accumulation to overcome Cd toxicity

**Figure 1:**
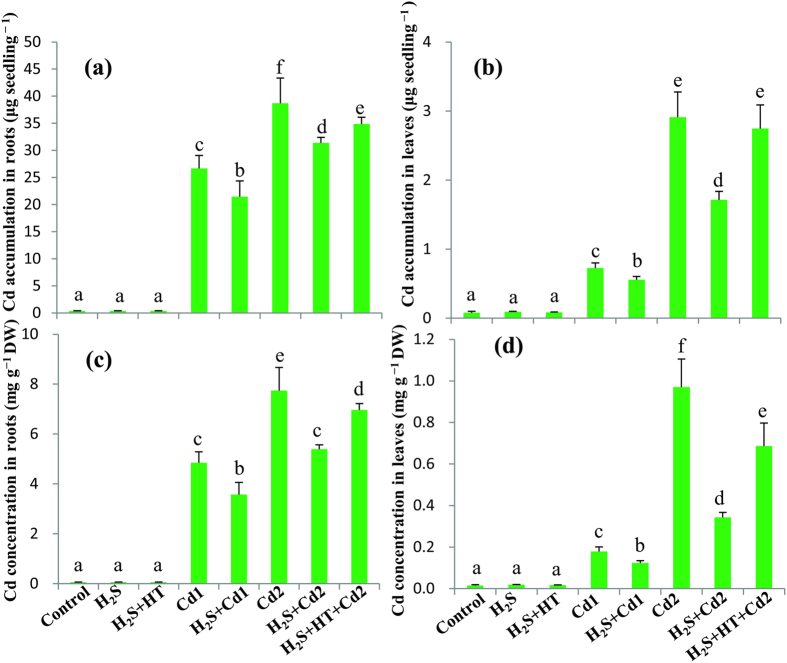
Effect of NaHS on Cd homeostasis in rice plants subjected to Cd stresses. (**a**) Cd accumulation in roots, (**b**) Cd accumulation in leaves, (**c**) Cd content in roots and (**d**) Cd content in leaves. Control, H_2_S, H_2_S + HT, Cd1, H_2_S + Cd1, Cd2, H_2_S + Cd2, and H_2_S + HT + Cd2 correspond to the group of seedlings exposed to only nutrients, 100 μM NaHS, 100 μM NaHS + 200 μM hypotaurine, 250 μM CdCl_2_, 100 μM NaHS + 250 μM CdCl_2_, 500 μM CdCl_2_, 100 μM NaHS + 500 μM CdCl_2_ and 100 μM NaHS + 200 μM hypotaurine + 500 μM CdCl_2_, respectively. Bars represent standard deviation (SD) of the mean (*n* = 3). Different letters (**a**–**f**) indicate significant differences among the treatments at *P* < 0.05, according to Duncan’s multiple range test. DW, dry weight.

The role of H_2_S in Cd homeostasis was investigated by measuring the levels of Cd in the roots and leaves of Cd-stressed rice plants supplemented with or without NaHS. Results in Fig. 1 showed that Cd uptake and accumulation increased in roots and leaves in a dose-dependent manner, and roots were the major organ in which most of the Cd deposited. H_2_S supplementation exhibited an inhibitory effect on Cd uptake and accumulation. Cd accumulation was decreased by 20 and 19% in roots and 23 and 41% in leaves of H_2_S + Cd1 and H_2_S + Cd2 groups, respectively, as compared with the corresponding Cd1 and Cd2 groups (Fig. 1a,b). As a consequence, Cd content was reduced by 26 and 30% in roots, and 30 and 65% in leaves of H_2_S + Cd1 and H_2_S + Cd2 groups (Fig. 1c,d). Furthermore, the H_2_S-mediated reduction in Cd uptake and accumulation was significantly minimized in NaHS and hypotaurine (HT)-treated H_2_S + HT + Cd2 group as compared with H_2_S + Cd2 group. These results together firmly demonstrated the regulatory role of H_2_S in Cd homeostasis.

### H_2_S harmonizes mineral nutrient balance under Cd stress

To provide an insight into the role of H_2_S in maintaining mineral homeostases under Cd stress conditions, we estimated the contents of Ca, Mg, Fe, Zn and Mn in both leaves and roots of rice plants (Table 1). In comparison with control group, the leaf contents of Ca, Mg, Fe, Zn and Mn were decreased by 23, 10, 13, 22 and 53% in Cd1 group and 23, 23, 61, 26 and 68% in Cd2 group, respectively. An increase in the contents of Ca (139 and 235%) and Fe (15 and 48%) but a decrease in the levels of Mg (22 and 25%), Zn (26 and 33%), and Mn (27 and 55%) was recorded in roots of Cd-treated plants (Cd1 and Cd2 groups), as compared with control group. Applying NaHS enhanced accumulation of Ca, Mg, Fe, Zn and Mn in H_2_S + Cd1 and H_2_S + Cd2 groups by 21 and 28, 8 and 19, 34 and 152, 28 and 47, and 44 and 217% in leaves; while in roots it enhanced accumulation of Mg, Fe and Zn in the same groups by 18 and 43, 13 and 14, and 73 and 60%, respectively, as compared with the corresponding Cd1 and Cd2 groups. Conversely, co-application of HT and NaHS disturbed mineral homeostases, as Ca, Mg, Fe, Zn and Mn contents were decreased by 13, 2, 49, 44 and 32%, respectively, in the leaves, whereas Mg, Fe and Zn levels were reduced by 31, 22, and 60%, respectively, in the roots of H_2_S + HT + Cd2 group when compared with H_2_S + Cd2 group (Table 1). Furthermore, NaHS addition significantly increased the contents of all these minerals in both roots and leaves of H_2_S group compared with control group.

### Exogenous H_2_S improves phenotypic appearance

**Figure 2:**
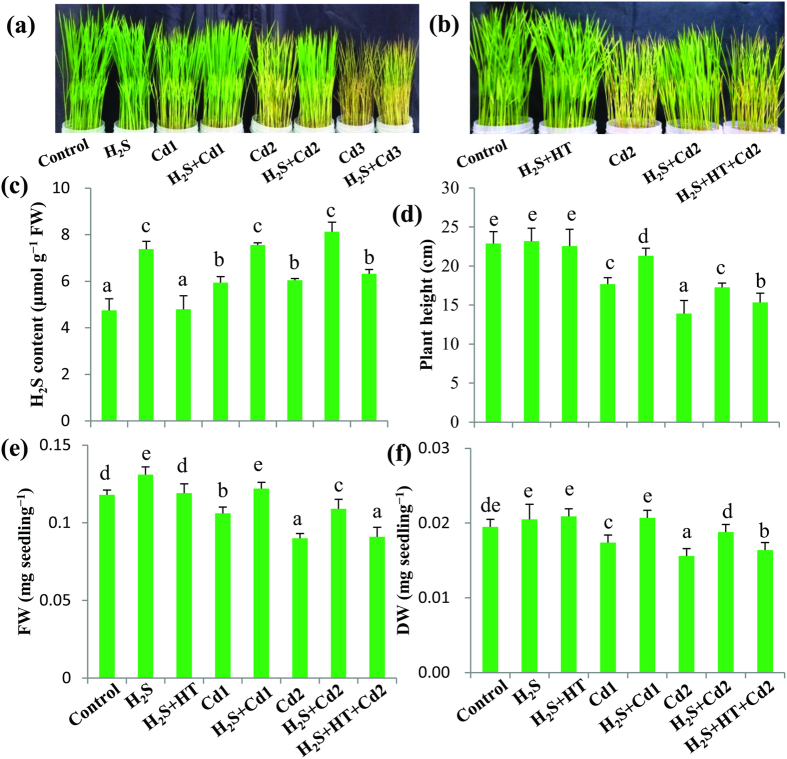
Effects of NaHS on phenotypes in the absence (**a**) and presence (**b**) of hypotaurine, endogenous level of H_2_S (**c**), plant height (**d**), fresh weight (**e**) and dry weight (**f**) of rice plants under Cd stress conditions. Control, H_2_S, H_2_S + HT, Cd1, H_2_S + Cd1, Cd2, H_2_S + Cd2, H_2_S + HT + Cd2, Cd3 and H_2_S + Cd3 correspond to the group of seedlings exposed to only nutrients, 100 μM NaHS, 100 μM NaHS + 200 μM hypotaurine, 250 μM CdCl_2_, 100 μM NaHS + 250 μM CdCl_2_, 500 μM CdCl_2_, 100 μM NaHS + 500 μM CdCl_2_, 100 μM NaHS + 200 μM hypotaurine + 500 μM CdCl_2,_ 1000 μM CdCl_2_ and 100 μM NaHS + 1000 μM CdCl_2_, respectively. FW, fresh weight; DW, dry weight. Bars represent standard deviation (SD) of the mean (n = 3). Different letters (a-e) indicate significant differences among the treatments at P < 0.05, according to Duncan’s multiple range test.

To investigate the possible effects of H_2_S in alleviating Cd toxicity, growing rice seedlings were initially exposed to three different concentrations of CdCl_2_ (250, 500 and 1000 μM) in the presence or absence of NaHS. Visual toxicity symptoms, including stunted growth, chlorosis and leaf rolling, gradually pronounced by Cd stress treatment in a Cd concentration-dependent manner, as compared with the untreated control (Fig. 2a). Exogenous NaHS almost nullified the toxicity symptoms in H_2_S + Cd1 group and those symptoms were drastically reduced in H_2_S + Cd2 group. However, the toxicity symptoms were most severe in Cd3 group and NaHS-mediated recovery was not very strong in H_2_S + Cd3 group to sustain normal growth. Therefore, the Cd3 and H_2_S + Cd3 groups were discontinued in further investigations. Application of HT, on the other hand, abolished the beneficial effects of H_2_S, as the toxicity symptoms in H_2_S + HT + Cd2 group were similar to that of Cd2 group, which implies that H_2_S released from NaHS was effective in alleviating Cd toxicity in rice plants (Fig. 2b). Moreover, no phenotypic variations were observed between control, H_2_S and H_2_S + HT groups, suggesting that NaHS itself or co-application of HT and NaHS did not exert any toxic effect on the phenotypes of the rice plants (Fig. 2a,b).

### Application of NaHS enhances endogenous level of H_2_S

In order to ascertain the beneficial effect of exogenous NaHS, we estimated the endogenous level of H_2_S in the presence and absence of Cd stresses. Results in Fig. 2c showed that NaHS application increased H_2_S level by 55% in H_2_S group but did not affect the level in H_2_S + HT group when compared with the untreated control. Cd stress also resulted in the increment of H_2_S content by 25 and 27% in Cd1 and Cd2 groups over the control level. Adding NaHS caused a further increase in the level of H_2_S by 27 and 35% in H_2_S + Cd1 and H_2_S + Cd2 groups, as compared with Cd1 and Cd2 groups, respectively. However, adding HT with NaHS decreased H_2_S level by 22% in H_2_S + HT + Cd2 group compared with H_2_S + Cd2 group, clearly indicating the increased level of endogenous H_2_S was attributed to exogenous NaHS. This elevated level of H_2_S is correlated with the enhanced Cd tolerance as reflected in the phenotypes of the rice plants treated with Cd and NaHS (Fig. 2a,b).

### Positive effects of H_2_S on growth and biomass

The effect of H_2_S on the growth parameters of rice plants under Cd stress were investigated in terms of plant height and biomass (FW and DW). As expected, plant height was reduced by 23 and 39% in Cd1 and Cd2 groups, respectively, as compared with control group (Fig. 2d). Application of NaHS with Cd reduced the negative effect of Cd stress on plant height, as it was recovered by 21 and 24% in H_2_S + Cd1 and H_2_S + Cd2 groups compared with Cd1 and Cd2 groups respectively. However, H_2_S-mediated plant height recovery was reduced by 11% in H_2_S + HT + Cd2 group when compared with H_2_S + Cd2 group (Fig. 2d). In comparison with control group, plant FW was decreased by 10 and 24%, and DW by 11 and 20% in Cd1 and Cd2 groups respectively. On the other hand, adding NaHS with Cd diminished the negative impact of Cd stress on plant biomass where FW was increased by 15 and 21%, and DW by 19 and 21% in H_2_S + Cd1 and H_2_S + Cd2 groups, as compared with Cd1 and Cd2 groups, respectively. Again, H_2_S-mediated plant biomass recovery in H_2_S + HT + Cd2 group was reduced by 17% (FW) and 13% (DW), as compared with H_2_S + Cd2 group (Fig. 2e,f), indicating that H_2_S scavenger HT negatively affected the plant biomass as well as plant growth recovery in Cd-stressed plants. Under non-stress conditions, plant height and DW remained constant in H_2_S and H_2_S + HT groups but FW was increased by 11% in H_2_S group, as compared with control group.

### H_2_S rescues the losses of Chl, carotenoids and water soluble protein contents

Cd toxicity caused a sharp decline in the levels of Chl *a* (16 and 45%), Chl *b* (20 and 43%), total Chl (17 and 45%), carotenoids (22 and 42%) and water soluble proteins (14 and 33%) in Cd1 and Cd2 groups in a Cd concentration-dependent manner, compared with control group (Table 2). The negative effects of Cd on the photosynthetic pigments, carotenoids and water soluble proteins were substantially minimized by exogenous NaHS. The levels of Chl *a*, Chl *b*, total Chl, carotenoids and soluble proteins increased by 16, 14, 15, 29 and 9% in H_2_S + Cd1 group and by 50, 32, 45, 38 and 19% in H_2_S + Cd2 group, respectively, as compared with their respective Cd1 and Cd2 groups (Table 2). Nevertheless, the H_2_S-mediated recovery on the losses of photosynthetic pigments and soluble protein contents in H_2_S + HT + Cd2 group was significantly reduced due to simultaneous application of NaHS and HT (Table 2). Water soluble proteins significantly declined in H_2_S + HT group but remained unchanged in H_2_S group compared with control group.

### H_2_S maintains water status and inhibits endogenous Pro accumulation

Leaf RWC was decreased by 17 and 26% in Cd1 and Cd2 groups, respectively, as compared with the untreated control (Table 2). However, the observed 17 and 19% increase in leaf RWC in H_2_S + Cd1 and H_2_S + Cd2 groups relative to that of Cd1 and Cd2 groups, respectively, indicated that exogenous NaHS significantly prevented the reduction of RWC in rice plants under Cd stress conditions. On the other hand, H_2_S-mediated restoration of leaf RWC significantly reversed in H_2_S + HT + Cd2 group (Table 2). Being an osmoprotectant, Pro accumulates in response to water shortage in plants under abiotic stresses, which might help plant adapt better to water deficit^33^. Cd stress resulted in a significant increase in Pro content in the leaves of Cd-stressed plants, which reached the maximum in Cd2 group (768%), when compared with the untreated control. NaHS addition decreased Pro content by 8 and 73% in H_2_S + Cd1 and H_2_S + Cd2 groups in comparison with Cd1 and Cd2 groups, respectively. However, simultaneous application of HT and NaHS increased Pro content by 159% in H_2_S + HT + Cd2 compared with H_2_S + Cd2 group, which coincided with the decrease in the level of RWC as well (Table 2). Under non-stress conditions, the levels of Pro and RWC remains unchanged in H_2_S and H_2_S + HT groups in comparison with the control group.

### H_2_S alleviates Cd-induced oxidative damage

H_2_O_2_ and lipid peroxidation product, MDA, are frequently used as major indicators of oxidative stress^16^. A drastic rise in the levels of H_2_O_2_ was recorded in Cd1 (40%) and Cd2 (75%) groups compared with the untreated control (Table 3). LOX, as an oxidative enzyme, often participates in lipid peroxidation contributing to oxidative stress^34^. LOX activity was also found to be significantly increased in Cd1 and Cd2 groups compared with control group. As a consequence, MDA level was markedly increased by 49 and 204% in Cd1 and Cd2 groups, respectively, when compared with control group (Table 3). On the other hand, adding NaHS reduced Cd-induced oxidative stress as evident by 26 and 29% decrease in H_2_O_2_ level, 21 and 29% decrease in LOX activity and a resultant decrease in MDA level by 30 and 42% in H_2_S + Cd1 and H_2_S + Cd2 groups, respectively, when compared with their respective Cd1 and Cd2 groups. Furthermore, co-application of HT and NaHS resumed oxidative stress in H_2_S + HT + Cd2 group as evident by a 35% increase in H_2_O_2_ level, a 29% increase in LOX activity and finally a 45% increase in MDA level as compared with H_2_S + Cd2 group (Table 3). Under non-stress conditions, H_2_O_2_ content increased by 12% only in H_2_S group, MDA content remained unchanged in both H_2_S and H_2_S + HT groups, and LOX activity increased by 24% only in H_2_S + HT group, as compared with control group.

### H_2_S maintains ROS homeostasis

**Figure 3:**
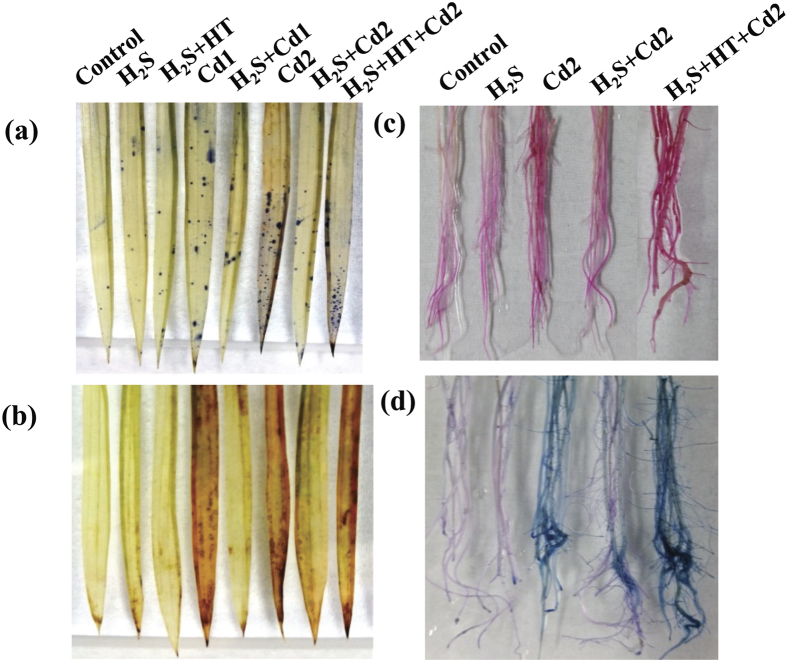
Histochemical detection of ROS accumulation in leaves, and lipid peroxidation and loss of membrane integrity in roots of rice plants under co-application of NaHS and CdCl_2_. (**a**) NBT staining of superoxide (O_2_^•^^−^), (**b**) DAB staining of hydrogen peroxide (H_2_O_2_), (**c**) Schiff’s test for lipid peroxidation and (**d**) Evan’s blue uptake test for loss of membrane integrity. Control, H_2_S, H_2_S + HT, Cd1, H_2_S + Cd1, Cd2, H_2_S + Cd2, and H_2_S + HT + Cd2 correspond to the group of seedlings exposed to only nutrients, 100 μM NaHS, 100 μM NaHS + 200 μM hypotaurine, 250 μM CdCl_2_, 100 μM NaHS + 250 μM CdCl_2_, 500 μM CdCl_2_, 100 μM NaHS + 500 μM CdCl_2_ and 100 μM NaHS + 200 μM hypotaurine + 500 μM CdCl_2_, respectively. NBT, nitroblue tetrazolium; DAB, diaminobenzidine.

To estimate the potential role of H_2_S in ROS homeostasis, we visualized the production of ROS like O_2_^•^^−^ and H_2_O_2_ in the leaves of rice plants with or without Cd stresses (Fig. 3a,b). Overaccumulation of O_2_^•^^−^ and H_2_O_2_ was visualized histochemically in a concentration-dependent manner in the leaves of rice plants subjected to Cd stresses. Conversely, NaHS application considerably diminished the accumulation of O_2_^•^^−^ and H_2_O_2_ in H_2_S + Cd1 and H_2_S + Cd2 groups, as compared with Cd1 and Cd2 groups, respectively, indicating a role for H_2_S in controlling ROS homeostasis in Cd-stressed plants. Additionally, scavenging H_2_S by HT in H_2_S + HT + Cd2 group caused an over-accumulation of O_2_^•^^−^ and H_2_O_2_, showing similar staining intensity to that of Cd2 group (Fig. 3a,b). Adding NaHS alone or in combination with HT in the absence of Cd stress did not alter the generation of O_2_^•^^−^ and H_2_O_2_ compared with the untreated control.

### Evidence of H_2_S-mediated protection of membrane integrity

Increased ROS production is supposed to disturb membrane integrity through lipid peroxidation^35^. Schiff’s test was employed to assess the ability of H_2_S to protect membrane from Cd-induced lipid peroxidation. Lipid peroxidation was considerably increased, as observed by intense red color, in the roots of rice plants under Cd stress when compared with the untreated control (Fig. 3c). However, NaHS application was found to reduce the lipid peroxidation in the roots of Cd-stressed rice plants, which is comparable to that of the untreated control. A parallel experiment about the staining of the roots with Evan’s blue was carried out to confirm the loss of membrane integrity due to lipid peroxidation. We observed that Cd stress resulted in an increased uptake of Evan’s blue, as indicated by deep blue color, by the roots of the rice plants under Cd stress alone (Fig. 3d). In contrast, adding NaHS with Cd considerably decreased the root uptake of Evan’s blue when compared with Cd stressed roots only. Moreover, application of NaHS and HT with Cd showed a severe disturbance in plasma membrane integrity as evidenced by higher stained intensity of roots (Fig. 3c,d). Collectively, these results suggest a healing effect of H_2_S for plasma membrane of rice roots under Cd stress conditions.

### H_2_S efficiently elevates AsA and GSH levels, and redox status

A noticeable reduction of AsA level (23 and 47% in Cd1 and Cd2 groups, respectively) and increment of DHA (17% in Cd2 group) were observed, as compared with the untreated control (Table 4). However, AsA/DHA ratio significantly decreased in these stress groups (Cd1 and Cd2). Adding NaHS increased AsA level by 22 and 38% and AsA/DHA ratio by 19 and 136% in H_2_S + Cd1 and H_2_S + Cd2 groups in comparison with Cd1 and Cd2 groups, respectively. However, AsA level decreased by 30%, DHA content increased by 103%, and AsA/DHA ratio decreased by 66% in H_2_S + HT + Cd2 group, as compared with H_2_S + Cd2 group. Although GSH content significantly increased in Cd1 group, it was drastically decreased by 32% in Cd2 group in comparison with the untreated control (Table 4). GSSG content increased by 23% and GSH/GSSG ratio decreased by 45% in Cd2 group compared with control group. NaHS addition did not affect GSH content in H_2_S + Cd1 group but it increased GSH content by 124% and GSH/GSSG ratio by 304% in H_2_S + Cd2 group as compared with Cd2 group (Table 4). Additionally, H_2_S-mediated increase in the level of GSH and GSH/GSSG ratio significantly reversed in H_2_S + HT + Cd2 group when compared with H_2_S + Cd2 group. Thus, H_2_S elevated the levels of AsA and GSH, and thus the redox status, to boost the antioxidant capacity of Cd-stressed rice plants.

### H_2_S upregulates various antioxidant enzymes to counteract oxidative stress

**Figure 4:**
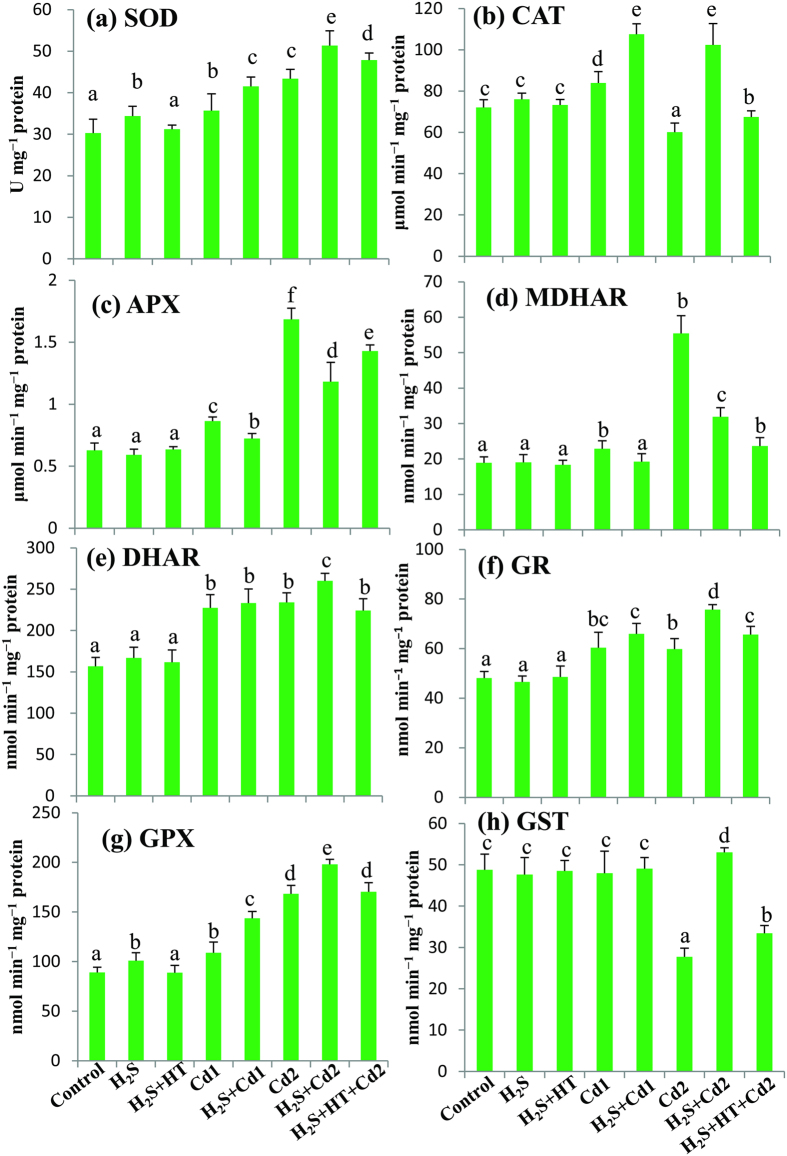
Effects of NaHS on the activities of ROS detoxifying enzymes in the leaves of rice plants with or without Cd stress. (**a**) superoxide dismutase (SOD), (**b**) catalase (CAT), (**c**) ascorbate peroxidase (APX), (**d**) monodehydroascorbate reductase (MDHAR), (**e**) dehydroascorbate reductase (DHAR), (**f**) glutathione reductase (GR), (**g**) glutathione peroxidase (GPX) and (**h**) glutathione *S*-transferase (GST). Control, H_2_S, H_2_S + HT, Cd1, H_2_S + Cd1, Cd2, H_2_S + Cd2, and H_2_S + HT + Cd2 correspond to the group of seedlings exposed to only nutrients, 100 μM NaHS, 100 μM NaHS + 200 μM hypotaurine, 250 μM CdCl_2_, 100 μM NaHS + 250 μM CdCl_2_, 500 μM CdCl_2_, 100 μM NaHS + 500 μM CdCl_2_ and 100 μM NaHS + 200 μM hypotaurine + 500 μM CdCl_2_, respectively. Bars represent standard deviation (SD) of the mean (*n* = 3). Different letters (**a**–**f**) indicate significant differences among the treatments at *P* < 0.05, according to Duncan’s multiple range test.

To explore the regulatory role of H_2_S in alleviation of Cd-induced oxidative stress, the activities of various antioxidant enzymes were investigated (Fig. 4a–h). SOD activity was increased by 18 and 43% in Cd1 and Cd2 groups, respectively, whereas CAT activity increased by 16% in Cd1 group and then declined by 17% under higher concentration of Cd (Cd2 group) as compared with control group. Applying H_2_S showed an increased in SOD (16 and 18%) and CAT (28 and 70%) activities in H_2_S + Cd1 and H_2_S + Cd2 groups, respectively, when compared with their respective Cd1 and Cd2 groups, which corroborates the levels of O_2_^•^^−^ and H_2_O_2_ (Figs 3a,b and 4a,b). As expected, combined application of NaHS and HT significantly decreased the activities of SOD and CAT in H_2_S + HT + Cd2 group when compared with H_2_S + Cd2 group.

The ascorbate-glutathione cycle contributes to reduce oxidative stress by detoxifying H_2_O_2_ through multistep enzymatic reactions^35^. APX and MDHAR activities increased in an intensity-dependent manner in the leaves of Cd-stressed rice plants (Fig. 4c,d). In comparison with the untreated control, DHAR and GR activities were increased by 45 and 26%, respectively, in Cd1 group but their activities did not increase further upon increasing the level of Cd concentration (Cd2 group) (Fig. 4e,f). Applying NaHS did not show boosting effect on APX and MDHAR activities but further increased DHAR (11%) and GR (27%) activities in H_2_S + Cd2 group relative to Cd2 group. Meanwhile, co-treatment of NaHS and HT caused a similar pattern of APX and MDHAR activities in Cd2 group but significantly curtailed the activities of DHAR and GR in H_2_S + HT + Cd2 group when compared with H_2_S + Cd2 group. No significant differences in the activities of APX, MDHAR, DHAR and GR were observed in NaHS and H_2_S + HT groups when compared with the untreated control (Fig. 4c–f).

GSH metabolizing enzymes GPX and GST showed differential responses under Cd stress; that is, GPX activity increased in a Cd concentration-dependent manner, whereas GST activity drastically decreased by 43% in Cd2 group in comparison with untreated control (Fig. 4g,h). Exogenously applied H_2_S exhibited a further increase in GPX activity by 32 and 18% in H_2_S + Cd1 and H_2_S + Cd2 groups, respectively, while it reduced the loss of GST activity in H_2_S + Cd2 group. Co-application of NaHS and HT significantly decreased the activities of GPX and GST in H_2_S + HT + Cd2 group when compared with H_2_S + Cd2 group. Following NaHS addition alone, GPX activity showed significant enhancement but GST activity remained unchanged in H_2_S group as compared with respective control groups.

### H_2_S attenuates MG toxicity by enhancing the activities of Gly enzymes

**Figure 5:**
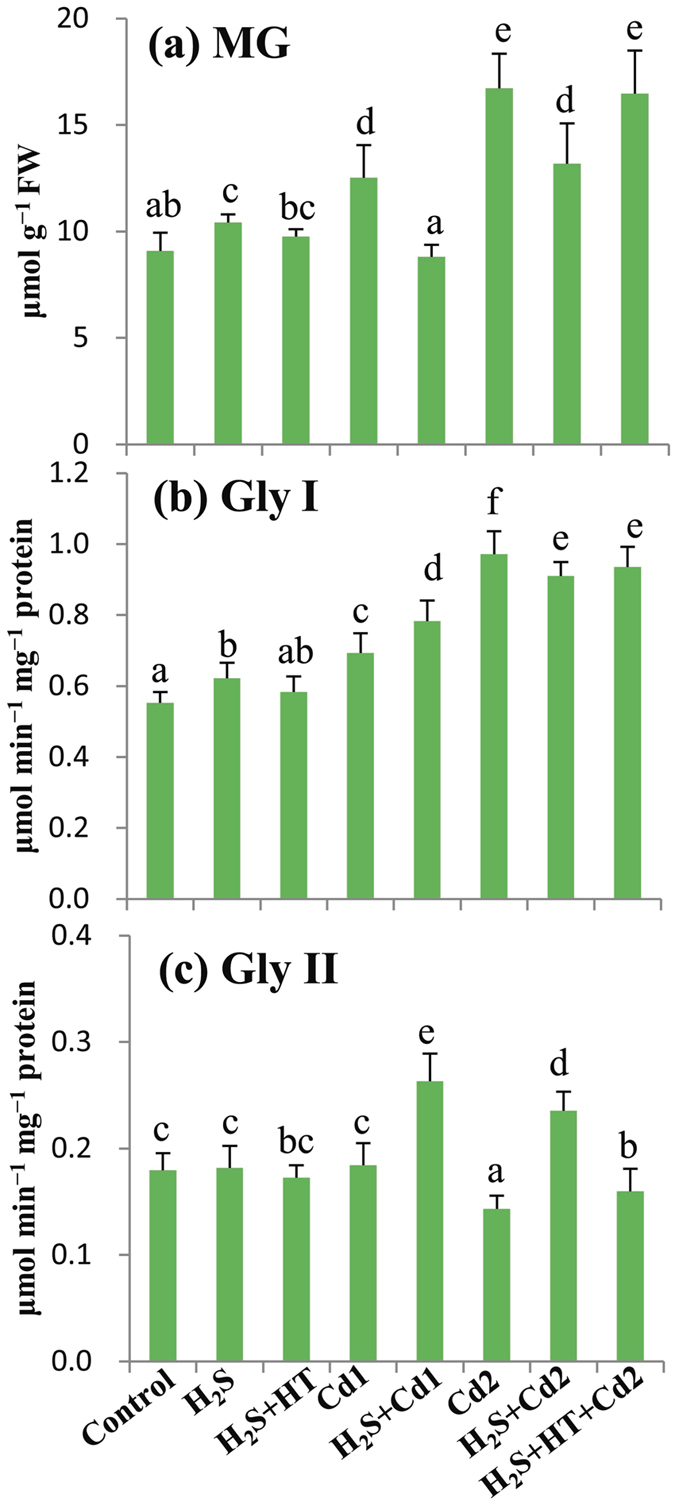
Effects of exogenous NaHS on methylglyoxal detoxification system in leaves of rice plants with or without Cd stress. (**a**) Methylglyoxal (MG) content, (**b**) Glyoxalase (Gly) I activity and (**c**) Gly II activity. Control, H_2_S, H_2_S + HT, Cd1, H_2_S + Cd1, Cd2, H_2_S + Cd2, and H_2_S + HT + Cd2 correspond to the group of seedlings exposed to only nutrients, 100 μM NaHS, 100 μM NaHS + 200 μM hypotaurine, 250 μM CdCl_2_, 100 μM NaHS + 250 μM CdCl_2_, 500 μM CdCl_2_, 100 μM NaHS + 500 μM CdCl_2_ and 100 μM NaHS + 200 μM hypotaurine + 500 μM CdCl_2_, respectively. Bars represent standard deviation (SD) of the mean (*n* = 3). Different letters (**a**–**f**) indicate significant differences among the treatments at *P* < 0.05, according to Duncan’s multiple range test. FW, fresh weight.

The reactive aldehyde MG is detoxified by the maintenance of GSH homeostasis via Gly enzymes^8^,^9^. MG level increased gradually as the Cd concentration increased; that is 38% in Cd1 group and 84% in Cd2 group, as compared with control group (Fig. 5a). A significant inhibition in MG production was observed in H_2_S + Cd1 and H_2_S + Cd2 groups when compared with Cd1 and Cd2 groups, respectively. Moreover, co-supplementation of NaHS and HT increased the level of MG by 25% in H_2_S + HT + Cd2 group compared with H_2_S + Cd2 group. In the presence or absence of NaHS, MG detoxifying enzymes exhibited differential responses in Cd-stressed seedlings (Fig. 5b,c). Gly I activity was increased by 25 and 76% in Cd1 and Cd2 groups, respectively, as compared with the untreated control. Applying NaHS resulted in an increase in Gly I activity by 13% in H_2_S + Cd1 group and a decrease by 6% in H_2_S + Cd2 group, as compared with Cd1 and Cd2 groups, respectively. Insignificant change in Gly II activity was observed in Cd1 group but the activity was reduced by 21% in Cd2 group relative to control group (Fig. 5c). NaHS addition showed noticeable increase in Gly II activity by 43 and 64% in H_2_S + Cd1 and H_2_S + Cd2 groups when compared with Cd1 and Cd2 groups, respectively. On the other hand, adding H_2_S scavenger HT displayed insignificant change in Gly I activity and a decrease in Gly II activity by 32% in H_2_S + HT + Cd2 group compared with H_2_S + Cd2 group (Fig. 5b,c).

## Discussion

**Figure 6:**
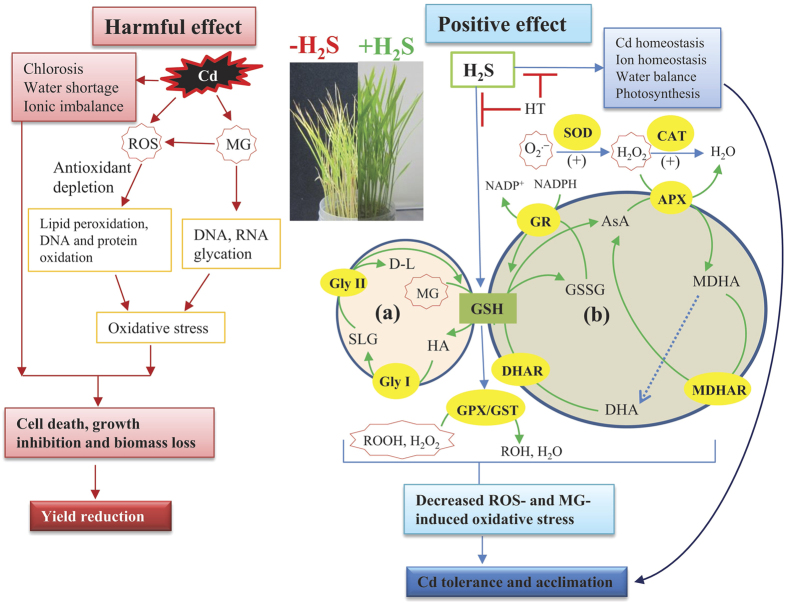
A Schematic diagram representing Cd-induced toxicity and protective mechanism of H_2_S underlying rice tolerance to Cd stress. Cd stress exerted harmful effects by inhibiting photosynthesis and affecting water status and ionic balance. Excessive Cd can induce reactive oxygen species (ROS) production by depleting antioxidant levels. Cd can also overaccumulate methylglyoxal (MG) that is highly toxic to DNA, RNA and proteins. ROS and MG contribute to oxidative stress, resulting in lipid peroxidation, DNA and protein oxidation, all of which cause growth inhibition, biomass loss and ultimate yield reduction. In contrast, elevated H_2_S level following sodium hydrosulfide (NaHS) application showed protective mechanism against Cd-induced toxicity by re-establishing Cd homeostasis, ionic balance and water balance, and by enhancing photosynthesis. H_2_S enhanced the detoxification of ROS by stimulating the activities of SOD and CAT. H_2_S also replenished the Cd-induced reduction in glutathione (GSH) level, which in turn enhanced the ROS and MG detoxifications as well as redox homeostases, leading to alleviation of oxidative stress. As a consequence, H_2_S enhanced Cd stress tolerance by retaining better growth of Cd-stressed rice plants. ( + ) indicates stimulatory effect of H_2_S. Dotted arrow indicates spontaneous conversion. Blunted arrow indicates inhibitory effect of hypotaurine (HT). (**a**,**b**) indicate Gly system and AsA-GSH system, respectively. AsA, ascorbic acid; DHA, dehydroascobate; GSSG, oxidized GSH; Gly, glyoxalase; HA, hemithioacetals; SLG, *S*-D-lactoylglutathione; D-L, D-lactic acid; O_2_^•^^−^, superoxide; H_2_O_2_, hydrogen peroxide; SOD, superoxide dismutase; CAT, catalase; APX, ascorbate peroxidase; MDHAR, monodehydroascorbate reductase; DHAR, dehydroascorbate reductase; GR, GSH reductase; GPX, GSH peroxidase; GST, GSH *S*-transferase; ROOH, lipid hydroperoxide; NADPH, nicotinamide adenine dinucleotide phosphate.

Among the various heavy metals, Cd is considered non-essential and highly toxic, adversely affecting growth, development and quality of lives of all living organisms, including plants^2^,^13^. Nowadays, rapid industrialization and excess use of fertilizers and pesticides in agriculture greatly contribute to the deposition of Cd in water and soils^2^,^4^,^14^. Once contamination occurred, Cd is readily accumulated in the aboveground parts of plants, thereby entering food chains and threatening human health worldwide^1^,^2^. Plant cells have evolved several defense strategies in restricting Cd toxicity, including (i) inhibition of uptake by exudates like organic acids, (ii) exclusion by sugar alcohols, (iii) chelation, conjugation and sequestration into vacuoles by strong ligands like GSH and phytochelatins and (iv) expression of anti-stress genes associated with thiol metabolism and antioxidant defense systems^1^,^8^,^14^. In this study, we have provided evidence that NaHS addition increased the level of endogenous H_2_S, which in turn modulates physiological and biochemical mechanisms associated with Cd stress tolerance in hydroponically raised rice plants. Furthermore, the alleviating effects of H_2_S were confirmed by adding the H_2_S scavenger HT, which dismounted the level of H_2_S and subsequently negated all the positive effect of H_2_S (Tables 1, 2, 3, 4 and Figs 1, 2, 3, 4, 5, 6).

Results indicated that Cd contents in the Cd-challenged rice plants increased in a Cd-concentration dependent manner, which was also correlated with the severity of the damage induced by excessive Cd (Figs 1 and 2a,b). Roots are the primary armaments that plants deploy to accumulate most of the heavy metals exposed^36^,^37^,^38^,^39^, as was also observed in this study. However, excessive Cd can have detrimental effects on root architecture, which affects plants’ capacity to absorb water and minerals^7^. Our results also showed that excessive Cd accumulation considerably reduced the uptake of several essential minerals in the roots and leaves of rice plants with the exception of Ca and Fe in the roots, whose content was increased upon increasing Cd concentrations (Table 1). More importantly, Cd stress significantly decreased the leaf contents of these minerals, indicating that the complex interactions between toxic and nutrient elements interfere with the mechanism of root-to-shoot transfer of these elements in the plants. In line with the finding of Ali *et al*.^32^, this result indicated that Cd-induced disturbance in mineral homeostasis hampered mineral-driven biochemical events in Cd-stressed rice plants. However, NaHS application significantly restricted the uptake and accumulation of Cd, thereby minimizing antagonistic effects of Cd on essential mineral transportation in rice plants (Fig. 1 and Table 1). It is plausible that co-application of CdCl_2_ and NaHS might produce non-toxic cadmium sulfide (CdS) to some extent, which partly contributed in decreasing cellular levels of Cd. This pattern of Cd homeostasis explained a specific mechanism that restored the capability of plants to accumulate essential nutrients for normal metabolic functions, and at the same time avoid Cd toxicity by keeping its level below the toxicity threshold (Fig. 6). This finding is supported by Dawood *et al*.^31^, who investigated mineral homeostases in *Hordeum vulgare* under the combined treatment of aluminum and NaHS.

In relation to the increased levels of Cd in the roots and leaves, growth and biomass of Cd-challenged rice plants in terms of plant height, FW and DW were greatly suppressed (Fig. 2d–f), as was reported in other plant species^38^,^39^. Cd-caused reduction in the level of the photosynthetic pigments and water soluble proteins also accorded with the reduced plant growth and biomass (Table 2 and Fig. 2), suggesting that Cd impaired photosynthetic ability by disrupting chloroplast structure, Chl-protein complexes and perhaps by deactivating the enzymes of Calvin cycle^5^,^40^. H_2_S, on the other hand, alleviated Cd-induced decline in Chl *a*, Chl *b*, carotenoids and proteins contents, thereby improving overall growth and biomass of Cd-stressed rice plants. The possible biochemical mechanism of H_2_S in improving rice growth could partly be attributed to the enhancement of Chl biosynthesis by delivering Mg, Fe, Zn and Mn ions and protection of Chl pigments from ROS by enhancing the level of carotenoids. Additionally, H_2_S, as a signaling molecule, can improve photosynthetic performance by promoting biogenesis of chloroplast and increasing the ability of CO_2_ fixation as observed in *Spinacea oleracea*^25^.

Plant-water imbalance and accumulation of osmolytes like Pro are general consequences of heavy metal toxicity, including Cd^33^,^38^. In this study, we found that leaf RWC and Pro levels were inversely related under Cd stress, whereas H_2_S addition restored RWC without accumulating much Pro (Table 2). These data suggested that H_2_S likely employed other metabolic adjustment(s) in which high level of Pro accumulation was not needed. Importantly, the observed enhancement of RWC (Table 2) might lead to an enhanced stomatal conductance and transpiration in H_2_S-treated Cd-stressed rice plants which in turn contributes to the improvement of photosynthesis^41^,^42^, thereby enhancing the overall performance of the rice plants.

In our study, the rice plants treated with Cd exhibited a severe oxidative stress in leaf tissues as evident by increased level of H_2_O_2_ and overproduction of ROS like O_2_^•^^−^ and H_2_O_2_ (Table 3 and Fig. 3a,b). This Cd-induced ROS together with increased LOX activity were correlated with the significant increase in MDA level (Table 3), indicating that Cd potentiated oxidative injuries by peroxidizing membrane lipids. Such effects were further confirmed by *in vivo* staining of peroxidation of lipid and injury of plasma membrane integrity in root tissues (Fig. 3c,d). Although Cd is unable to generate ROS via Haber–Weiss or Fenton reactions, it can induce ROS production, and thus oxidative stress, by increasing the activity of plasma membrane NADPH oxidases, depleting the non-enzymatic antioxidants, inhibiting/down- regulating ROS-detoxifying enzymes and disrupting electron transport chain^43^,^44^. Plants have evolutionally equipped with ROS-detoxifying techniques and the enhanced productions and synchronized actions of various antioxidants govern the balance between ROS generation and detoxification in plant cells under stress conditions^16^,^17^,^36^,^45^. The current study revealed that Cd, particularly at higher concentrations, seriously impaired ROS detoxification mechanism by decreasing the contents of AsA and GSH, as well as the redox states in terms of AsA/DHA and GSH/GSSG ratios (Table 4 and Fig. 6). Moreover, unsynchronized activities of SOD and CAT (Fig. 4a,b), which constitute the frontline enzymatic network by converting O_2_^•^^−^ and H_2_O_2_ consecutively into H_2_O^16^, further accelerated the accumulation of ROS in Cd-stressed rice plants (Fig. 3a,b). Importantly, we observed that CAT activity increased at lower concentration, but significantly decreased at higher concentration of Cd. Similar results were obtained in different plant species, including rice under Cd stress^38^,^41^,^46^,^47^,^48^. The possible mechanism might be that at low concentration, Cd stimulates CAT activity to enhance the basal antioxidant capacity to overcome oxidative stress. However, at higher concentration, overaccumulation of Cd inhibits CAT activity, possibly by replacing the Fe from the active center of CAT^7^. It was also suggested that Cd applied to plants at higher doses and/or for longer exposure period inhibits the synthesis of CAT or downregulates CAT activity or inactivates CAT in plants, mainly by disturbing the assembly of different subunits of CAT^49^. On the other hand, H_2_S-induced amendment of AsA and GSH levels and redox states as well as enhancement of SOD and CAT activities (Table 4 and Figs 3a–d and 4a,b) augmented the detoxification of ROS, thereby demonstrating an efficient role of H_2_S in tailoring major antioxidant defense system to protect the cells from oxidative damage.

The ascorbate-glutathione cycle involves a series of reactions with four enzymes (APX, MDHAR, DHAR and GR) that act coordinately in removing H_2_O_2_ and maintaining cellular redox balance, especially under stress conditions^16^,^17^,^35^. In this study, despite the stimulation of the activities of all four enzymes upon Cd stress, the H_2_O_2_ level remained significantly higher (Table 3 and Fig. 4c–f), which indicated that their stimulation was not up to the requisite level in lowering excessive H_2_O_2_. In consistent with our findings, Dixit *et al*.^19^ also observed an accumulation of H_2_O_2_ even after increasing the activity of H_2_O_2_ scavenging enzymes in roots and leaves of *Pisum sativum*, suggesting that production of H_2_O_2_ exceeded ROS scavenging capacity. In contrast, H_2_S, by intensifying DHAR and GR activities as well as maintaining APX and MDHAR activities above the control level, coordinates the activities of these four enzymes in the presence of enhanced SOD and CAT activities, thereby contributing well to the regulation of ROS level (Figs 4a-f, 3a,b, 6 and Table 3). This antioxidative role of H_2_S supported by the observation that H_2_S increased the activity of antioxidant enzymes, including SOD, CAT, DHAR and GR and the abundance of their corresponding transcripts, which were responsible for enhanced salt tolerance in *Medicago sativa*^28^. Interestingly, we observed that NaHS addition increased H_2_O_2_ content by 12% under non-stress condition; however, MDA content remained unchanged. This finding indicated that the increase in H_2_S level did not exert oxidative stress (Table 3). Thus, we assumed that this slight increase in the amount of H_2_O_2_ might stimulate the signaling role of H_2_S, as H_2_O_2_ is a well-known signaling molecule with positive regulatory role at lower cellular concentration^50^.

Another important mechanism in regulating heavy metal-induced toxicity is associated with the GSH-dependent conjugation of lipid hydroperoxides and endobiotic substrates by GPX and GST^8^,^18^. Our findings indicated that higher DHAR and GPX activities together with inefficient recycling of GSSG by GR might have contributed to the Cd-induced depletion of GSH and GSH/GSSG ratio, which consequently distressed the activity of GST (Fig. 4e–h and Table 4). However, a careful analysis of GSH-related defense mechanism under H_2_S addition revealed that H_2_S replenished the depleted GSH level, possibly by inducing its biosynthesis or by upregulating GR activity under Cd stress, which then participates in redox homeostasis as well as GPX and GST-mediated efficient scavenging of peroxides and other electrophiles (Fig. 6). This finding together with reduced LOX activity (Table 3) suggested a membrane protecting role of H_2_S under Cd stress in rice plants. Our results are consistent with those reported by Cui *et al*.^51^, who found that H_2_S-induced GSH and ROS homeostases were involved in enhancing alfalfa tolerance to Cd stress.

Additionally, the current study showed encouraging results on plant defense against MG toxicity under combined application of NaHS and Cd. Several reports^25^,^28^,^29^,^31^, including this study, proved the regulatory role of H_2_S on antioxidant defense; however, its role in GSH-based MG detoxification has remained to be determined in plant systems. We noticed a correlation between increased MG levels and inefficient Gly system in Cd-stressed plants, as the activity of Gly II decreased upon increasing the concentration of Cd (Fig. 5a–c), thereby intensifying Cd toxicity in rice plants. On the other hand, the observed lower contents of MG in H_2_S- and Cd-treated plants were associated with enhanced activities of Gly I and Gly II, which indicated a positive effect of H_2_S in amplifying MG detoxification (Fig. 6). This finding also suggested that enhanced Gly II activity efficiently recycled GSH into the system, which facilitated GSH homeostasis and higher activities of DHAR, GPX and GST (Table 4 and Figs 4f–h, 6) in preventing oxidative stress. It has also been reported that the enhanced activities of Gly enzymes and antioxidant enzymes (APX, GST and GPX) provided higher tolerance to Zn toxicity in *Brassica oleracea*^34^. Thus, our results suggested that H_2_S coordinates the biochemical actions of the antioxidant defense and Gly systems to mitigate the Cd-induced ROS and MG toxicity in rice plants (Fig. 6).

Based on all the above observations, a schematic diagram was depicted in Fig. 6, which illustrates the physiological and biochemical mechanisms involving Cd-induced toxicity and the H_2_S-mediated Cd stress tolerance in rice plants. Our results provided strong evidence that H_2_S successfully alleviated Cd-induced growth inhibition and biomass loss, which was mainly attributed to (i) decreased Cd uptake and accumulation, (ii) enhanced mineral homeostases and photosynthetic pigments and (iii) reduced oxidative stress through inducing ROS and MG detoxifications by maintaining redox homeostases. Taking into account that heavy metal tolerance requires a coordination of complex physiological and biochemical processes, this study, therefore, advances our understanding of the complex system integrating rice responses to Cd stress, and perhaps other environmental stresses. However, molecular approaches using H_2_S-biosynthetic mutants should be considered in future studies to decipher the exact role of H_2_S in modulating tolerance mechanism in crop plants under Cd stress.

## Methods

### Plant materials, growth conditions and treatments

The germination and cultivation of rice (*Oryza sativa* L. cv. BRRI dhan52) in a hydroponic condition were carried out according to the method described by Mostofa *et al*.^52^,^53^ and Supplementary Fig. S1 online. The hydroponic culture consisted of a hyponex solution, which contains 8% N, 6.43% P, 20.94% K, 8% S, 11.8% Ca, 3.08% Mg, 0.07% B, 0.24% Fe, 0.03% Mn, 0.0014% Mo, 0.008% Zn, and 0.003% Cu. The hyponex solution was diluted to 5,000 fold and renewed every three days. Uniformly grown 13-d-old seedlings were exposed to nutrient solution supplemented with 250, 500 and 1000 μM of CdCl_2_ in the presence and absence of H_2_S donor sodium hydrosulfide (NaHS, 100 μM). These environmentally relevant CdCl_2_ concentrations were selected based on previous reports^4^,^46^. To verify the role of H_2_S released from NaHS, we applied H_2_S scavenger hypotaurine (HT, 200 μM)^28^,^47^ with 500 μM CdCl_2_ and 100 μM NaHS. Based on literature^32^,^47^,^48^ and our preliminary experiments with a range of NaHS concentrations (25, 50, 100, 150 and 200 μM), we observed that 100 μM NaHS was optimally effective in alleviating Cd-induced toxic symptoms. We also observed that 100 μM NaHS was not effective in alleviating the toxic effects of high dose of CdCl_2_ (1000 μM CdCl_2_) (Fig. 1a). Therefore, our experimental design consisted of eight treatments as follows: (i) Control, (ii) 100 μM NaHS (H_2_S), (iii) 100 μM NaHS + 200 μM hypotaurine (H_2_S + HT), (iv) 250 μM CdCl_2_ (Cd1), (v) 100 μM NaHS + 250 μM CdCl_2_ (H_2_S + Cd1), (vi) 500 μM CdCl_2_ (Cd2), (vii) 100 μM NaHS + 500 μM CdCl_2_ (H_2_S + Cd2) and (viii) 100 μM NaHS + 200 μM hypotaurine + 500 μM CdCl_2_ (H_2_S + HT + Cd2) (see Supplementary Table S1 online). The seedlings were harvested after 3 days of Cd stress treatments to investigate the H_2_S-induced mechanisms modulating physiological and biochemical responses. Each treatment was replicated three times under the same experimental conditions.

### Growth parameters

The growth of rice seedlings was assessed by measuring plant height, fresh weight (FW) and dry weight (DW). Plant height was determined by measuring the length from the bottom of the main stem to the end of the emerging third leaf. To determine FW and DW, 10 seedlings from each treatment were weighed and oven-dried at 80 °C for 48 h, then expressed as g seedling^−1^.

### Determination of H_2_S content

H_2_S content in rice leaves was determined following the method described by Christou *et al*.^54^ with modifications. Rice leaves (0.25 g) were homogenized in 1 mL of 100 mM K-P buffer (pH 7.0) containing 10 mM EDTA and centrifuged at 11,200 × *g* for 15 min. The supernatant (100 μL) was mixed with 1,880 μL extraction buffer and 20 μL of 20 mM 5,5′-dithiobis (2-nitrobenzoic acid) and incubated at 25 °C for 5 min. The absorbance was read at 412 nm, and H_2_S was quantified using a standard curve developed with known concentrations of NaHS.

### Measurement of Cd and mineral nutrient contents in roots and leaves

To determine Cd and mineral nutrient contents (Ca, Mg, Fe, Zn and Mn), the root and leaf samples were harvested separately and oven-dried at 80 °C for 48 h. Dried samples (0.1 g) were ground and digested with a HNO_3_:HClO_4_ (5:1 v/v) mixture at 80 °C until the yellow color disappeared. The Cd, Ca, Mg, Fe, Zn and Mn contents were measured by using flame atomic absorption spectrophotometry.

### Relative water content and proline content

Relative water content (RWC) was determined as described by Mostofa *et al*.^52^. Pro content was determined according to the method of Bates *et al*.^55^ with minor modifications as reported in Mostofa and Fujita^36^.

### Photosynthetic pigments

After extracting 0.5 g leaves in 80% chilled acetone, the absorbance of the supernatants was recorded at 663, 645 and 470 nm for determining the contents of Chl and carotenoids according to the formulas suggested by Arnon^56^ and Litchtentaler and Wellburn^57^, respectively.

Chl *a* content (mg g^−1^ FW) = (0.0127  ×  D663–0.00269  ×  D645) × V/W

Chl *b* content (mg g^−1^ FW) = (0.0229  ×  D645–0.00468  ×  D663) × V/W

Total Chl content (mg g^−1^ FW) = (20.2 × D645 + 8.02 × D663) × V/(1000 × W)

Carotenoid content (mg g^−1^ FW) = (1000  ×  A470–2.270  ×  Chl *a*—81.4  ×  Chl *b*/227) × V × 1000/W;where V = volume of 80% (v/v) acetone (mL), W = fresh weight of sample (g).

### Lipid peroxidation and H_2_O_2_ content

Lipid peroxidation of the leaves was measured by estimating the MDA content according to the method of Heath and Packer^58^. H_2_O_2_ was extracted and determined after reaction with 0.1% TiCl_4_ in 20% H_2_SO_4_ following the method of Mostofa *et al*.^53^.

### Histochemical analyses

For detecting lipid peroxidation and the loss of membrane integrity in roots, Schiff’s reagent test and Evan’s blue test were performed, respectively, following the method of Lai *et al*.^28^. Histochemical analyses of two major ROS molecules O_2_^•^^−^ and H_2_O_2_ were carried out in rice leaves following the method of Mostofa and Fujita^36^.

### Extraction and estimation of non-enzymatic antioxidants

Fresh leaves (0.5 g) were homogenized in 3 mL of ice-cold 5% meta-phosphoric acid containing 1 mM EDTA and centrifuged at 11,500 × *g* for 15 min. Reduced and total AsA contents were assayed at 265 nm in 100 mM K-phosphate buffer (pH 7.0) with 1.0 U of ascorbate oxidase (AO) following the method of Mostofa and Fujita^36^. Oxidized ascorbate (DHA; dehydroascorbic acid) content was calculated by deducting reduced AsA amount from total AsA content. Based on enzymatic recycling, total GSH and oxidized glutathione (GSSG) contents were determined according to the method of Griffiths^59^. Reduced GSH content was measured after subtracting the value of GSSG from total GSH level.

### Extraction and assay of enzymes

Extraction of enzymes was carried out following the method of Mostofa *et al*.^52^,^53^. Activities of antioxidant and glyoxalase enzymes were determined by the standard methods reported in Doderer *et al*.^60^ for LOX (EC 1.13.11.12), Mostofa and Fujita^36^ for SOD (EC 1.15.1.1) and CAT (EC 1.11.1.6), Nakano and Asada^61^ for APX (EC 1.11.1.11) and DHAR (EC 1.8.5.1), Hossain *et al*.^62^ for MDHAR (EC 1.6.5.4), Mostofa *et al*.^45^ for GR (EC 1.6.4.2), GST (EC 2.5.1.18) and GPX (EC: 1.11.1.9), Hossain *et al*.^63^ (2009) for Gly I (EC 4.4.1.5) and Mostofa and Fujita^36^ for Gly II (EC 3.1.2.6), respectively. Protein content was determined following the method of Bradford^64^ using bovine serum albumin (BSA) as a standard.

### Estimation of MG content

Following the method of Wild *et al*.^65^, leaves (0.5 g) were homogenized in 5 mL of 0.5 M perchloric acid and incubated for 15 min on ice. The extract was centrifuged for 10 min at 11,200 × *g* and 1 mL supernatant was transferred to a microcentrifuge tube containing 10 mg charcoal and kept at room temperature for 15 min. After centrifugation for 10 min at 11,200 × *g*, the supernatant was neutralized by saturated K_2_CO_3_ and then centrifuged at 11,200 × *g* for 10 min. The neutralized supernatant (650 μL), 100 mM potassium phosphate (K-P) buffer (pH 7.0, 330 μL) and 0.5 M N-acetyl-L-cysteine (20 μL) were added together and incubated for 15 min. Formation of N-α-acetyl-S- (1-hydroxy-2-oxo-prop-1-yl) cysteine was recorded at a wavelength of 288 nm and MG content was calculated by using a standard curve of known concentrations of MG.

### Statistical analysis

The data were subjected to one-way analysis of variance (ANOVA) and different letters indicate significant differences between treatments at *P* < 0.05, according to Duncan’s multiple range test (DMRT) using IRRISTAT version 3 (IRRI, Biometrics Unit, Manila, Philippines). Data represented in the Tables and Figures are means ± standard deviations (SD) of three replicates for each treatment.

## Additional Information

**How to cite this article**: Mostofa, M. G. *et al*. Hydrogen sulfide modulates cadmium-induced physiological and biochemical responses to alleviate cadmium toxicity in rice. *Sci. Rep*. **5**, 14078; doi: 10.1038/srep14078 (2015).

## Supplementary Material

Supplementary Information

## Figures and Tables

**Table 1:** Effects of exogenous NaHS on the levels of minerals in the leaves and roots of rice plants with or without Cd stress.

Treatments	Ca (mg g^−1^DW)		Mg (mg g^−1^DW)		Fe (mg g^−1^DW)		Zn (mg g^−1^DW)		Mn (mg g^−1^DW)	
	Leaves	Roots	Leaves	Roots	Leaves	Roots	Leaves	Roots	Leaves	Roots
Control	12.35 ± 0.41^d^	2.05 ± 0.12^a^	56.88 ± 1.27^cd^	24.96 ± 0.31^d^	0.70 ± 0.01^d^	2.66 ± 0.04^a^	0.23 ± 0.02^c^	0.15 ± 0.02^c^	0.19 ± 0.01^d^	0.11 ± 0.01^d^
H_2_S	15.65 ± 0.82^e^	3.06 ± 0.23^c^	65.91 ± 1.88^e^	35.54 ± 1.16^g^	0.80 ± 0.12^e^	2.94 ± 0.05^b^	0.30 ± 0.07^e^	0.25 ± 0.01^f^	0.33 ± 0.01^f^	0.16 ± 0.01^e^
H_2_S + HT	12.20 ± 0.51^cd^	2.60 ± 0.09^b^	58.65 ± 1.27^d^	27.52 ± 0.62^f^	0.82 ± 0.03^e^	2.50 ± 0.11^a^	0.19 ± 0.01^b^	0.18 ± 0.03^e^	0.22 ± 0.02^e^	0.10 ± 0.02^c^
Cd1	9.55 ± 0.56^a^	4.89 ± 0.38^e^	50.91 ± 3.70^b^	19.38 ± 0.33^b^	0.61 ± 0.03^c^	3.05 ± 0.11^b^	0.18 ± 0.02^b^	0.11 ± 0.02^b^	0.09 ± 0.02^b^	0.08 ± 0.01^b^
H_2_S + Cd1	11.55 ± 1.54^c^	3.37 ± 0.11^d^	54.98 ± 5.79^c^	22.84 ± 2.20^c^	0.82 ± 0.02^e^	3.46 ± 0.10^c^	0.23 ± 0.01^c^	0.18 ± 0.03^de^	0.13 ± 0.03^c^	0.08 ± 0.02^b^
Cd2	9.48 ± 0.84^a^	6.87 ± 0.82^g^	43.67 ± 2.41^a^	18.61 ± 2.23^ab^	0.27 ± 0.01^a^	3.95 ± 0.07^e^	0.17 ± 0.04^b^	0.10 ± 0.03^a^	0.06 ± 0.01^a^	0.05 ± 0.01^a^
H_2_S + Cd2	12.18 ± 2.27^cd^	5.35 ± 0.70^f^	52.15 ± 2.32^b^	26.54 ± 1.75^e^	0.68 ± 0.02^d^	4.52 ± 0.11^b^	0.25 ± 0.03^d^	0.16 ± 0.03^d^	0.19 ± 0.02^d^	0.04 ± 0.01^a^
H_2_S + HT + Cd2	10.54 ± 1.21^b^	5.29 ± 0.36^f^	50.98 ± 3.80^b^	18.40 ± 0.84^a^	0.35 ± 0.01^b^	3.53 ± 0.02^d^	0.14 ± 0.02^a^	0.10 ± 0.02^ab^	0.13 ± 0.01^c^	0.04 ± 0.01^a^

Control, H_2_S, H_2_S + HT, Cd1, H_2_S + Cd1, Cd2, H_2_S + Cd2, and H_2_S + HT + Cd2 correspond to the group of seedlings exposed to only nutrients, 100 μM NaHS, 100 μM NaHS + 200 μM hypotaurine, 250 μM CdCl_2_, 100 μM NaHS + 250 μM CdCl_2_, 500 μM CdCl_2_, 100 μM NaHS + 500 μM CdCl_2_ and 100 μM NaHS + 200 μM hypotaurine + 500 μM CdCl_2_, respectively. Values are means ± SD of three independent replications (n = 3). Different subscripted letters (a-g) within the column indicate statistically significant differences among the treatments according to Duncan’s multiple range test (*P < *0.05). DW, dry weight.

**Table 2:** Effects of NaHS on the levels of chlorophyll (Chl) *a*, *b*, total Chl, carotenoids, water soluble proteins, relative water content (RWC) and proline (Pro) in rice plants with or without Cd stress.

Treatments	Chl *a* (mg g^−1^ FW)	Chl *b* (mg g^−1^ FW)	Total Chl (mg g^−1^ FW)	Carotenoids (mg g^−1^ FW)	Water-soluble proteins (mg g^−1^FW)	RWC (%)	Pro (μmol g^−1^FW)
Control	2.75 ± 0.13^cd^	0.858 ± 0.14^f^	3.61 ± 0.19^e^	0.713 ± 0.02^d^	21.26 ± 1.16^e^	98.24 ± 0.31^e^	0.152 ± 0.02^a^
H_2_S	2.96 ± 0.09^d^	0.841 ± 0.07^f^	3.81 ± 0.17^f^	0.722 ± 0.03^d^	21.72 ± 1.48^e^	97.84 ± 0.43^e^	0.176 ± 0.01^ab^
H_2_S + HT	2.80 ± 0.14^d^	0.856 ± 0.05^f^	3.66 ± 0.14^e^	0.709 ± 0.01^d^	19.84 ± 1.66^d^	98.16 ± 0.17^e^	0.149 ± 0.02^a^
Cd1	2.31 ± 0.27^b^	0.686 ± 0.05^c^	3.00 ± 0.28^c^	0.553 ± 0.06^b^	18.38 ± 0.89^cd^	81.05 ± 0.72^b^	0.239 ± 0.01^c^
H_2_S + Cd1	2.67 ± 0.09^c^	0.785 ± 0.03^e^	3.45 ± 0.11^d^	0.714 ± 0.08^d^	19.46 ± 1.14^d^	94.45 ± 1.06^d^	0.214 ± 0.01^bc^
Cd2	1.50 ± 0.14^a^	0.490 ± 0.08^a^	1.99 ± 0.18^a^	0.481 ± 0.06^a^	14.30 ± 0.40^a^	72.49 ± 1.67^a^	1.319 ± 0.17^f^
H_2_S + Cd2	2.24 ± 0.25^b^	0.645 ± 0.07^d^	2.88 ± 0.32^c^	0.663 ± 0.08^c^	17.02 ± 0.93^bc^	86.26 ± 0.82^c^	0.355 ± 0.06^d^
H_2_S + HT + Cd2	1.57 ± 0.08^a^	0.499 ± 0.08^b^	2.07 ± 0.16^b^	0.499 ± 0.03^a^	16.18 ± 0.98^b^	73.05 ± 2.47^a^	0.921 ± 0.08^e^

Control, H_2_S, H_2_S + HT, Cd1, H_2_S + Cd1, Cd2, H_2_S + Cd2, and H_2_S + HT + Cd2 correspond to the group of seedlings exposed to only nutrients, 100 μM NaHS, 100 μM NaHS + 200 μM hypotaurine, 250 μM CdCl_2_, 100 μM NaHS + 250 μM CdCl_2_, 500 μM CdCl_2_, 100 μM NaHS + 500 μM CdCl_2_ and 100 μM NaHS + 200 μM hypotaurine + 500 μM CdCl_2_, respectively. Values are means ± SD of three independent replications (n = 3). Different subscripted letters (a, b, c, d, e and f) within the column indicate statistically significant differences among the treatments according to Duncan’s multiple range test (*P < *0.05). FW, fresh weight.

**Table 3:** Effects of NaHS on the levels of hydrogen peroxide (H_2_O_2_), malondialdehyde (MDA) and lipoxygenase (LOX) activity in rice plants with or without Cd stress.

Treatments	H_2_O_2_(nmol g^−1^ FW)	MDA (nmol g^−1^ FW)	LOX (nmol min^−1^ mg^−1^ protein)
Control	35.46 ± 1.73^a^	27.00 ± 1.95^a^	18.42 ± 1.19^a^
H_2_S	39.85 ± 0.90^b^	26.95 ± 1.73^a^	17.83 ± 1.47^a^
H_2_S + HT	35.06 ± 2.37^a^	28.26 ± 2.36^a^	22.78 ± 1.20^c^
Cd1	49.62 ± 3.92^d^	40.36 ± 1.84^b^	27.12 ± 2.46^d^
H_2_S + Cd1	36.82 ± 4.21^a^	28.12 ± 1.96^a^	21.32 ± 0.79^b^
Cd2	61.96 ± 4.47^f^	82.17 ± 6.51^e^	37.06 ± 2.35^f^
H_2_S + Cd2	43.95 ± 3.10^c^	47.76 ± 1.70^c^	26.33 ± 1.94^d^
H_2_S + HT + Cd2	59.40 ± 4.62^e^	69.20 ± 2.54^d^	34.07 ± 3.46^e^

Control, H_2_S, H_2_S + HT, Cd1, H_2_S + Cd1, Cd2, H_2_S + Cd2, and H_2_S + HT + Cd2 correspond to the group of seedlings exposed to only nutrients, 100 μM NaHS, 100 μM NaHS + 200 μM hypotaurine, 250 μM CdCl_2_, 100 μM NaHS + 250 μM CdCl_2_, 500 μM CdCl_2_, 100 μM NaHS + 500 μM CdCl_2_ and 100 μM NaHS + 200 μM hypotaurine + 500 μM CdCl_2_, respectively. Values are means ± SD of three independent replications (n = 3). Different subscripted letters (a-f) within the column indicate statistically significant differences among the treatments according to Duncan’s multiple range test (*P < *0.05). FW, fresh weight.

**Table 4:** Effects of NaHS on the levels of non-enzymatic antioxidants ascorbic acid (AsA) and glutathione (GSH), as well as their redox states (AsA/DHA and GSH/GSSG) in rice plants with or without Cd stress.

Treatments	AsA (nmol g^−1^FW)	DHA (nmol g^−1^FW)	AsA/DHA ratio	GSH (nmol g^−1^FW)	GSSG (nmolg^−1^FW)	GSH/GSSG ratio
Control	4883.87 ± 480.77^ef^	738.39 ± 42.88^d^	6.60 ± 0.27^c^	416.69 ± 32.55^b^	33.06 ± 2.87^c^	12.72 ± 2.10^bc^
H_2_S	4974.19 ± 194.53^f^	767.62 ± 75.33^d^	6.48 ± 0.49^c^	470.50 ± 30.07^c^	34.31 ± 1.49^c^	13.71 ± 0.32^c^
H_2_S + HT	4824.72 ± 52.43^e^	692.09 ± 52.40^c^	7.15 ± 1.24^d^	433.32 ± 16.49^b^	37.07 ± 2.76^d^	11.72 ± 0.71^b^
Cd1	3756.02 ± 180.91^c^	601.18 ± 61.55^b^	6.29 ± 0.55^c^	580.29 ± 41.89^d^	30.27 ± 2.90^b^	19.39 ± 3.77^d^
H_2_S + Cd1	4593.66 ± 110.01^d^	621.18 ± 67.45^b^	7.47 ± 0.97^d^	583.77 ± 9.51^d^	22.91 ± 1.81^a^	25.60 ± 2.25^e^
Cd2	2579.57 ± 202.56^a^	866.13 ± 40.52^e^	2.98 ± 0.11^b^	281.48 ± 34.26^a^	40.59 ± 2.36^e^	6.98 ± 1.60^a^
H_2_S + Cd2	3563.66 ± 81.11^b^	508.92 ± 53.84^a^	7.04 ± 0.58^d^	631.05 ± 35.43^e^	22.47 ± 1.89^a^	28.21 ± 2.68^f^
H_2_S + HT + Cd2	2493.32 ± 204.77^a^	1032.89 ± 27.93^f^	2.42 ± 0.26^a^	259.04 ± 38.67^a^	32.88 ± 2.35^c^	7.92 ± 1.50^a^

Control, H_2_S, H_2_S + HT, Cd1, H_2_S + Cd1, Cd2, H_2_S + Cd2, and H_2_S + HT + Cd2 correspond to the group of seedlings exposed to only nutrients, 100 μM NaHS, 100 μM NaHS + 200 μM hypotaurine, 250 μM CdCl_2_, 100 μM NaHS + 250 μM CdCl_2_, 500 μM CdCl_2_, 100 μM NaHS + 500 μM CdCl_2_ and 100 μM NaHS + 200 μM hypotaurine + 500 μM CdCl_2_, respectively. Values are means ± SD of three independent replications (n = 3). Different subscripted letters (a-f) within the column indicate statistically significant differences among the treatments according to Duncan’s multiple range test (*P < *0.05). FW, fresh weight; DHA, dehydroascorbic acid; GSSG, oxidized glutathione.
