# Supplementary material for: Hydrogen sulfide modulates cadmium-induced physiological and biochemical responses to alleviate cadmium toxicity in rice
Source: Sci Rep. 2015 Sep 11;5:14078. doi: 10.1038/srep14078 (PMC4566128; doi:10.1038/srep14078)
Supplement: Supplementary Information [file srep14078-s1.pdf]

Title: Hydrogen sulfide modulates cadmium-induced physiological and biochemical responses to alleviate cadmium toxicity in rice

Authors: Mohammad Golam Mostofa[1, 2], Anisur Rahman[1], Md. Mesbah Uddin Ansary[3], Ayaka Watanabe [1], Masayuki Fujita[1,]***,** and Lam-Son Phan Tran[4,]*

Supplementary Figure S1. Experimental set up. Healthy rice seeds were washed with distilled water and imbibed for 2 days in the dark. The imbibed seeds were sown on plastic nets floating on distilled water in 250 mL plastic beakers and kept in the dark for germination at 28 ± 2°C for 3 days. Uniformly germinated seeds were transferred to a growth chamber and grown in a commercial hydroponics solution. At 13 days, rice plants were subjected to a combination of different doses of NaHS, hypotaurine and $CdCl_2$. After 3 days of treatments, that is, at day 16th, plants were harvested for collecting data on various physiological and biochemical parameters.

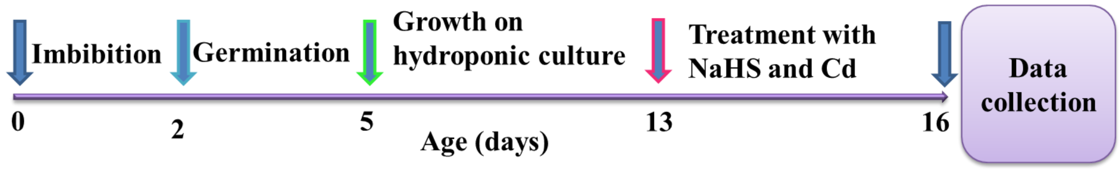

Supplementary Table S1. Experimental design.

No.	Treatments
1) Control	Only nutrient solution
2) H_2S	100 µM NaHS
3) H_2S+HT	100 µM NaHS + 200 µM hypotaurine
4) Cd1	250 µM $CdCl_2$
5) H_2S+Cd1	100 µM NaHS + 250 µM $CdCl_2$
6) Cd2	500 µM $CdCl_2$
7) H_2S+Cd2	100 µM NaHS + 500 µM $CdCl_2$
8) H_2S+ HT+Cd2	100 µM NaHS + 200 µM hypotaurine + 500 µM $CdCl_2$
9) Cd3	1000 µM $CdCl_2$
10) H_2S+Cd3	100 µM NaHS + 1000 µM $CdCl_2$

All the treatments were imposed by applying them in the nutrient solution. Control, H_2S, H_2S+HT, Cd1, H_2S+Cd1, Cd2, H_2S+Cd2, H_2S+HT+Cd2, Cd3 and H_2S+Cd3 correspond to the group of seedlings exposed to only nutrients, 100 µM NaHS, 100 µM NaHS + 200 µM hypotaurine, 250 µM $CdCl_2$, 100 µM NaHS + 250 µM $CdCl_2$, 500 µM $CdCl_2$, 100 µM NaHS + 500 µM $CdCl_2$, 100 µM NaHS + 200 µM hypotaurine + 500 µM $CdCl_{2,}$ 1000 µM $CdCl_2$ and 100 µM NaHS + 1000 µM $CdCl_2$, respectively.
